# Supplementary material for: Epidemiological Patterns of Seasonal Respiratory Viruses during the COVID-19 Pandemic in Madagascar, March 2020–May 2022
Source: Viruses. 2022 Dec 20;15(1):12. doi: 10.3390/v15010012 (PMC9864023; doi:10.3390/v15010012)
Supplement: Supplementary file 1 [file viruses-15-00012-s001.zip › viruses-2055564-supplementary.pdf]

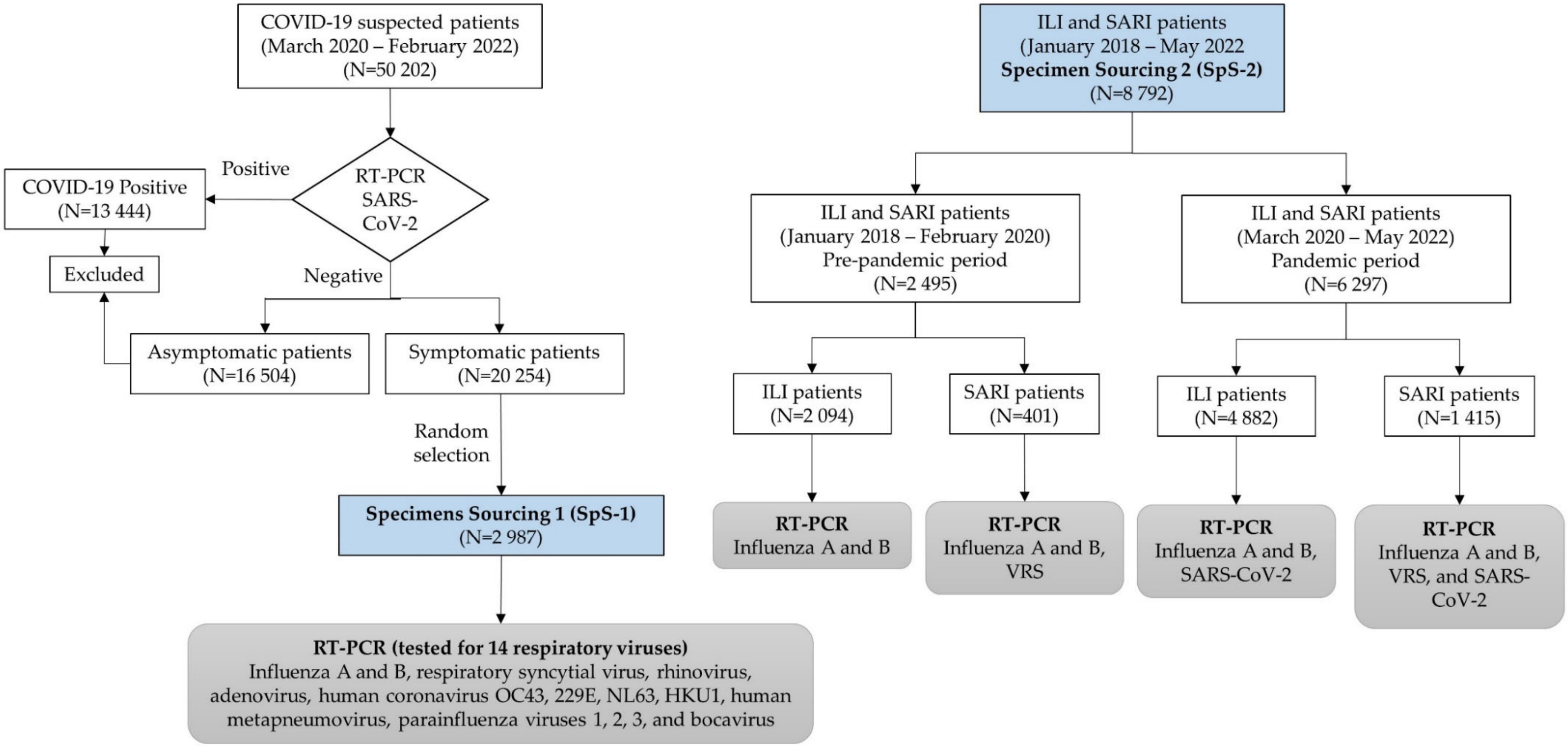

Figure S1. Description of the different source of specimens used in the study and tests performed accordingly.

Table S1. List of seasonal respiratory virus co-detected in patients during the three epidemic waves of SARS-CoV-2 in Madagascar.

Wave N°	Co-infections	N
1	HRV + CoV-OC43 + PIV2	3
	HRV + BOV	2
	HRV + CoV-OC43	2
	CoV-OC43 + PIV3	2
	CoV-OC43 + RSV	1
	CoV-OC43 + HMPV	1
	HMPV + CoV-NL63	1
	HMPV + PIV2	1
	PIV3 + CoV-229E	1
	PIV3 + ADV	1
2	HRV + RSV	3
	HRV + BOV	1
	HRV + CoV-229E	1
	HRV + HMPV	1
	RSV + BOV	1
	RSV + PIV2	1
	RSV + CoV-229E	1
	RSV + ADV	1
	HMPV + CoV-229E	1
	HMPV + BOV	1
	PIV2 + BOV	1
	CoV-OC43 + HMPV + ADV	1
3	HRV + RSV	2
	HRV + FLU B	1
	HRV + ADV	1
	HRV + BOV	1
	HRV + PIV1	1
	ADV + HMPV	1
	ADV + PIV1	1
	ADV + FLUB	1
	ADV + FLUA	1
	FLUA + CoV-NL63	1
	FLUA + HRV + ADV	1
	FLUA + BOV + ADV	1
	HRV + PIV1 + HMPV + ADV	1

ADV=Adenovirus; BOV=Bocavirus; CoV=Coronavirus; FLU=Influenza; HMPV=Human metapneumovirus; HRV=Human rhinoviruses; PIV=Parainfluenza viruses; RSV=Respiratory syncytial viruses.

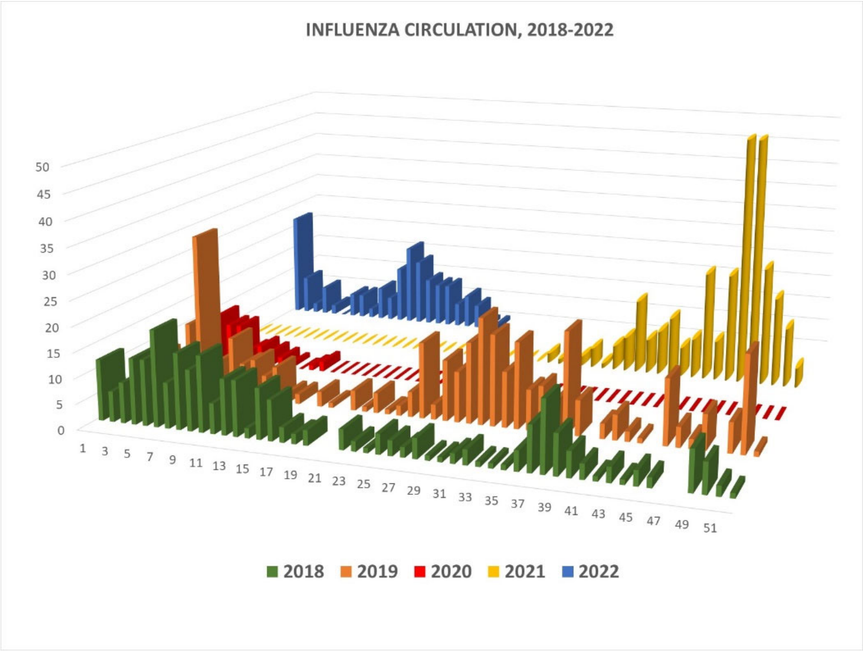

Figure S2. Weekly distribution of influenza detected from the influenza-like illnesses (ILI) Surveillance Network, Madagascar, January 2018 to May 2022.

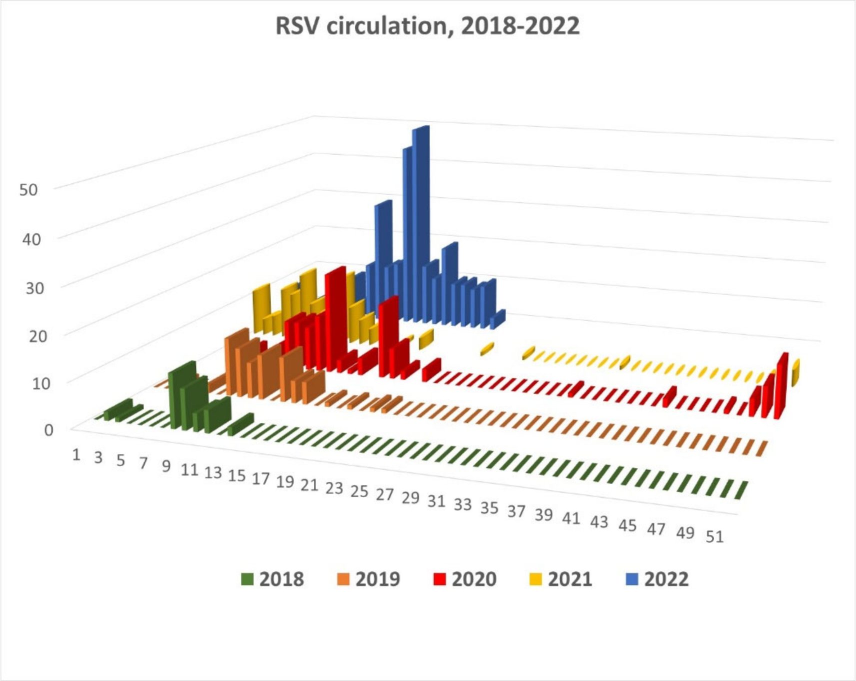

Figure S3. Weekly distribution of respiratory syncytial viruses (RSV) detected from severe acute respiratory infections (SARI) surveillance, Madagascar, January 2018 to May 2022.
